# Role of Long-Acting Injectable Second-Generation Antipsychotics in the Treatment of First-Episode Schizophrenia: A Clinical Perspective

**DOI:** 10.1155/2012/764769

**Published:** 2012-05-07

**Authors:** Radovan Přikryl, Hana Přikrylová Kučerová, Michaela Vrzalová, Eva Češková

**Affiliations:** ^1^Central European Institute of Technology, Masaryk University (CEITEC-MU), Kamenice 753/5, 62500 Brno, Czech Republic; ^2^Department of Psychiatry, Faculty of Medicine Masaryk University, University Hospital Brno, Jihlavska 20, 62500 Brno, Czech Republic

## Abstract

Approximately 80% of patients with the first-episode schizophrenia reach symptomatic remission after antipsychotic therapy. However, within two years most of them relapse, mainly due to low levels of insight into the illness and nonadherence to their oral medication. Therefore, although the formal data available is limited, many experts recommend prescribing long-acting injectable second-generation antipsychotics (mostly risperidone or alternatively paliperidone) in the early stages of schizophrenia, particularly in patients who have benefited from the original oral molecule in the past and agree to receive long-term injectable treatment. Early application of long-acting injectable second-generation antipsychotics can significantly reduce the risk of relapse in the future and thus improve not only the social and working potential of patients with schizophrenia but also their quality of life.

## 1. Depot Antipsychotics for the Treatment of Schizophrenia

First-generation long-acting injectable (depot) antipsychotics (AP1G) emerged in clinical practice in the 1960s. For the treatment of schizophrenia, their use resulted in a significant decrease in the number of patients relapses, including length and frequency of hospitalizations [1]. However, when oral second-generation antipsychotics (AP2G) were introduced thirty years later, the position (in clinical practice) of depot AP1G dramatically changed. Despite the benefits of AP1G, psychiatrists prescribed them for long-term therapy of schizophrenia much less often and started to switch to oral AP2G as they were seen as more efficient and better tolerated [2]. This trend persisted for many years, despite evidence to suggest from meta-analyses and naturalistic studies that depot AP1G were more effective in reducing schizophrenic relapses than oral AP2G [3]. The same finding was later logically replicated also for long-acting injectable (LAI) AP2G [4–7]. Although patients with schizophrenia are often willing to use depot or LAI antipsychotics, these preparations are today prescribed only for approximately 20% of them [8–10]. In a survey [11] psychiatrists answered that they only offer long-acting injectable antipsychotics to one in every three patients with schizophrenia.

## 2. Specifics for First-Episode Schizophrenia Therapy

Therapy for the first episode of schizophrenia has certain specific features. Patients respond to low doses of antipsychotics relatively well; yet they are more sensitive to the adverse effects, particularly extrapyramidal ones [12, 13]. Patients with first-episode schizophrenia, however, typically show low willingness to use antipsychotics in the long-term treatment, likely due to their high unawareness of the disease severity. Recommended duration of antipsychotic administration after the first episode of schizophrenia usually ranges from 1 to 2 years [12–14]. A five-year observation study of first-episode patients showed that the risk of relapse is five times higher after discontinuation of therapy compared to continuous medication [15]. Despite the recommendations from their psychiatrists, patients with schizophrenia discontinue their therapy often. Results of the clinical antipsychotic trials of intervention effectiveness (CATIE) study indicate that up to 74% of patients with schizophrenia discontinued their therapy after 18 months; the European First Episode Schizophrenia Trial (EUFEST) shows that up to 42% of patients terminated their treatment within one year after the disease onset [16, 17]. When considering the length of subsequent prophylactic treatment after the first episode of schizophrenia; not only its efficacy but also the profile or severity of adverse events of the given antipsychotic must be taken into account. Moreover, it should be taken into consideration that approximately 20% of first-episode patients will never experience a subsequent exacerbation of schizophrenia, irrespective of whether they receive or the type of therapy they receive [14, 18].

## 3. Problems Associated with Nonadherence to Pharmacological Treatment of Schizophrenia

Schizophrenia is a chronic mental disease characterized among other things by a high degree of nonadherence to prescribed medication [19]. It has been reported that patients take on average only 58% of their prescribed drugs, 41.2% of patients do not use their medication according to prescription, and one- to two-thirds of patients take their pills irregularly [20–22]. These are the results of questionnaire surveys, and therefore they reflect only the situation of patients that agreed to take part in the research; in reality, therefore the rates of nonadherence may be much higher. In general, the current guidelines on schizophrenia treatment consider depot or long-acting injectable antipsychotics as drugs of choice for long-term therapy in patients who are nonadherent with antipsychotic medication [12–14]. Systematic surveys of specific studies indicate that patients treated with depot antipsychotics show only a 24% non-adherence rate while they are sufficiently covered by medication for 91% of the total therapy time [23–25].

## 4. Long-Acting Injectable versus Oral Antipsychotics in the Treatment of Schizophrenia

Since available studies differ in methodology and primary observation goals [26], it is difficult to generalize on the effects of oral or long-acting injectable administration of antipsychotics on the course of schizophrenia. In a meta-analysis, Adams et al. [27] did not prove dominance of depot AP1G over oral antipsychotics in terms of reduction of the number of relapses. Nevertheless, overall improvement was seen more often in patients treated with depot antipsychotics [27]. On the other hand, a recent meta-analysis [7] found lower levels of relapse in patients with schizophrenia treated with depot AP1G or LAI AP2G, compared with those treated with oral antipsychotics. Still, the two therapeutic strategies did not differ in the number of rehospitalizations, terminations of therapy, or cases of non-adherence. Inconsistency of results might be explained by the nature of double-blind randomized studies that, of course, do not typically enroll non-adherent patients who are likely to benefit most from the depot/LAI formulation of antipsychotic therapy. Conditions of real clinical practice are therefore better simulated by observation studies where depot and oral antipsychotics are often compared with a mirror design. These studies describe almost consistently lower number of days of hospitalization during depot or LAI antipsychotic therapy, as compared with the same period of oral medication [1, 28]. The main methodological problem of these studies lies in the fact that, when the initial oral antipsychotic medication fails, a new oral treatment is prescribed, whilst the injectable medication remains the same. This can significantly affect the comparison of the therapy efficacy in identical time periods [26].

## 5. Long-Acting Injectable Antipsychotics in First-Episode Schizophrenia

Depot AP1Gs, let alone LAI AP2G, are rarely prescribed for patients with first-episode schizophrenia, although their levels of non-adherence and hence the risk of subsequent relapse are in reality very high [29, 30]. Importantly, adverse course of early schizophrenia has a profound negative influence on psychosocial integration of patients, not to mention a patients ability to remain in education and/or employment. Economic consequences of schizophrenia therefore represent a burden not only for health system but also for the social system of public health insurance. Psychiatrists usually explain the low prescription rate of LAI antipsychotics in the initial/early stage of schizophrenia treatment as an unwillingness of patient to receive injections on an outpatient basis or generally by negative attitudes to depot/LAI antipsychotics [31, 32].

## 6. Long-Acting Injectable Antipsychotics in First-Episode Schizophrenia: Attitudes of Psychiatrists

Heres et al. [33] used a questionnaire to ask almost 200 German psychiatrists at the national congress in 2008 about their attitudes towards the use of LAI antipsychotics in patients with first-episode schizophrenia. They found that long-term injectable therapy was only offered to 26.7% of patients with first-episode schizophrenia; it was actually prescribed to 13.3% of those offered (i.e., one of every two). Respondents also reported that up to 60.4% patients taking antipsychotics irregularly will relapse within one year after the first episode [33]. The main outcome of this questionnaire was that it identified the three principal reasons for *not* using LAI antipsychotics in patients with first-episode schizophrenia. German psychiatrists stated that they find it difficult to present the benefits of a long-term injectable antipsychotic therapy to patients who do not have any personal experience with schizophrenia relapse, as they are still at the very beginning of their illness. Poor availability of LAI AP2G was identified as another reason, particularly when AP2G are strongly preferred over AP1G, especially in patients with first-episode schizophrenia. The last reason was mainly personal reservations of the psychiatrists associated associated with difficult control of adverse effects of depot antipsychotics, negative impact on patient-psychiatrist relationship, or higher amount of psychiatrist's time needed to manage depot medication administration [33]. The survey indicates that the only barrier to more frequent use of LAI AP2G in patients with first-episode schizophrenia, which is not influenced by perceptions of patients and psychiatrists, is the issue with market availability, price, and method of reimbursement. At present, only LAI risperidone, olanzapine pamoate, and paliperidone palmitate are available in most countries. For many reasons, limited availability of these drugs is today considered as the main barrier of their more common use in the therapy of schizophrenia [2, 11, 32]. This is problem mainly in first-episode patients in whom AP2G are strongly preferred as drugs of first choice [13, 34, 35]. Hopefully, better availability of LAI AP2G, simpler prescription rules, and better reimbursement from public health insurance will result in more common use of these products in clinical practice. However, this logical sequence of thoughts is in conflict with the real situation in the UK where better availability of LAI risperidone did not increase the use of LAI antipsychotics for treatment of schizophrenia despite previous belief of British psychiatrists that availability of these products was highly desirable and would significantly change their prescription habits in favor of LAI antipsychotics [2, 32]. Keeping this in mind, the latter assumption that better availability of LAI AP2G would make psychiatrists prescribe LAI preparations more often sounds ironic [32]. Other reasons for not prescribing LAIs reflect subjective attitudes of psychiatrists and patients towards injectable therapy in general. The questionnaire by Heres et al. clearly highlighted that only one in four patients with first-episode schizophrenia was offered the possibility of treatment with LAI AP2G and half of those that were, agreed. That means that three out of four patients were not offered this type of therapy from their psychiatrist. Actually, the fact that psychiatrists, driven by their own personal negative attitudes/perceptions, offer LAI AP2G less frequently is a true barrier to using these products more commonly in patients with first-episode schizophrenia. Psychiatrists often say that first-episode patients reject LAI AP2G because they have not yet experienced a schizophrenic relapse. In this sense, higher efficacy of a LAI AP2G in prevention of relapses may not be a feasible argument [1, 5, 36]. Alternatively, low prescription rates can be explained by the fact that psychiatrists suppose beforehand (without discussing the issue with patients) that first-episode patients will not be interested in LAI AP2G therapy for the above-mentioned reasons. Interestingly, high self-confidence of psychiatrists to foreknow attitudes of their patients is considered to be one of the key factors of less frequent use of LAI AP2G in clinical practice [2, 32]. 

## 7. Long-Acting Injectable Antipsychotics in First-Episode Schizophrenia: Current Knowledge

Recent studies show that long-term injectable AP2G therapy is effective and also acceptable for first-episode patients, which is in opposition to the above-mentioned “conservative” attitudes [30, 37]. Unfortunately, studies comparing long-term injection or depot therapy with oral antipsychotic treatment after the first episode of schizophrenia are rare [38]. From this perspective, unique data can be drawn from a nation wide cohort study aimed at identification of the risk of rehospitalization and therapy discontinuation in more than 2,500 patients first hospitalized for schizophrenia between 2000 and 2007 in Finland [26]. The results show that in Finland, where antipsychotics are fully reimbursed from general health insurance systems, only 45.7% patients pick up prescribed drugs within one month and continue their therapy. Data are highly representative, since the study used the national registry and incorporated every single patient in Finland who was hospitalized for schizophrenia for the first time. Although information about medication during hospitalization was not available, almost all patients were recommended to start subsequent antipsychotic therapy. As compared with studies such as CATIE [16] or EUFEST [17], the Finnish study indicated higher rate of therapy discontinuation. This could be explained by the nature of the study which made it possible to capture real everyday behavior of patients, irrespective of their motivations or willingness to cooperate. As Finland, just as the majority of other countries, has not established any compulsory outpatient psychiatric screening, comparison of efficiency of individual antipsychotics can be generalized only for persons who were willing to visit their psychiatrist on an outpatient basis. It turned out that administration of depot AP1G and LAI risperidone resulted in reduction of the risk of rehospitalization by 50% or 65%, respectively, compared with oral formulations of the same antipsychotics. Depot AP1G or LAI risperidone were the first-choice drug in 8% of patients, and, in total, they comprised 10% of treated patient-years [26]. This is relatively low, especially when compared with patients with chronic schizophrenia. So far, these antipsychotic formulations have been earmarked mainly for patients with low insight into the illness and poor adherence to the therapy. However, if the target population for LAI AP2G would be extended to include also patients with better insight and apparent adherence, rehospitalization rate should decrease. Of course, this would apply only to patients who are willing to use these products on an outpatient basis.

## 8. Long-Acting Injectable Second-Generation Antipsychotics in First-Episode Schizophrenia

As far as LAI AP2G products are concerned, researchers have clinical experience with LAI risperidone, olanzapine pamoate, and paliperidone palmitate. Although many studies in schizophrenia with these products have been performed, relatively limited data on their use specifically in first-episode patients is available. Only LAI risperidone has been specifically studied for the use in early stage of schizophrenia (see Table 1) in addition specific post-hoc analyses have been performed in patients classified as having recent onset schizophrenia. Parellada et al. [39] designed a six-month open-label study with LAI risperidone to analyze a subgroup of 382 patients with recent schizophrenia or schizoaffective disorder (diagnosed ≤ 3 years ago) [40]. Schizophrenia was diagnosed in 84% of patients, with median of one year after the diagnosis was established. Previous medication included mainly AP2G (70%) and depot AP1G (24%). Non-adherence (42%) and poor efficacy (31%) of previous medication were the main reasons for changing the therapy. The study was completed by 73% patients who showed significant decrease of severity of schizophrenic symptomatology, reflected by statistically significant reduction of not only total PANSS score (positive and negative syndrom scale) [41] but also all PANSS subscales. In 40% of patients, total PANSS score decreased by at least 20%. At the same time, patients showed an improvement of overall functioning, quality of life, and satisfaction [39]. In other study, Emsley et al. [30] administered LAI risperidone monotherapy to fifty patients with the first episode of schizophrenia: this two-year observation study was completed by 36 patients (72%), 39 patients (78%) showed reduction of symptoms by at least 50%; 4 of them relapsed, 32 patients (64%) reached remission according to the proposed criteria for the remission in schizophrenia [42], and 31 patients (62%) reached remission that persisted throughout the two years of the study [30]. After two years, 33 patients decided to terminate the therapy. Seventy-nine percent (79%) of those then subsequently relapsed (median to relapse: 163 days). Based on these results, Emsley et al. [43] assert that first-episode patients who reached remission are prone to relapse after discontinuation of continuous long-acting injectable risperidone therapy. Antipsychotic treatment in such patients should therefore be continuous and maintained for at least two years [43]. Malla et al. [44] published data from their two-year prospective open multicentric study that was performed with young patients (aged 18 to 30 years) suffering from schizophreniform disorder, schizophrenia, or schizoaffective disorder (for no longer than 3 years). Patients were randomized to treatment with oral AP2G or LAI risperidone. Although it is difficult to generalize the results due to low number of subjects enrolled in the study (*n* = 15), patients treated with LAI risperidone showed more significant reduction of total PANSS score (by 16.1), as compared with oral AP2G (by 5.0) [44]. Weiden et al. [37] published preliminary data about early adherence from their randomized controlled study that compared LAI risperidone with oral AP2G in first-episode patients [37]. Nineteen (19, 73%) of 26 patients who were asked to participate in the study agreed to be treated with LAI risperidone; the other group consisted of 11 patients. Adherence rate in the twelfth week of observation was comparable in both groups. However, adherence questionnaires indicated that patients accepting LAI risperidone therapy showed higher probability of adherence, as compared with patients treated with oral antipsychotics [37]. Bartzokis et al. [45] examined the impact of antipsychotic formulation on the myelination trajectory during a randomized six-month trial of LAI risperidone versus oral risperidone in first-episode schizophrenia subjects. Two groups (11 patients treated with LAI risperidone and 13 patients treated with oral risperidone) that were matched in prerandomization oral medication exposure and 14 healthy controls were prospectively examined. Frontal lobe white matter volume was estimated using inversion recovery MRI (magnetic resonance imaging) images. A brief neuropsychological battery that focused on reaction times was performed at the end of the study. White matter volume remained stable in the LAI risperidone group and decreased significantly in the oral risperidone group resulting in a significant differential treatment effect, while the healthy controls had a white matter change intermediate and not significantly different from the two schizophrenia groups. White matter increase was associated with faster reaction times in tests involving frontal lobe function. The results suggest that LAI risperidone may improve the trajectory of myelination in first-episode patients and has a beneficial impact on cognitive performance. Better adherence provided by LAI AP2G may underlie the modified trajectory of myelin development [45].

## 9. Benefits of Long-Acting Injectable Antipsychotics in First-Episode Patients with Schizophrenia: Clinical Perspective and Summary

LAI AP2G have been used in clinical practice for several years. Nowadays, LAI AP2G are reserved mainly for patients with long-term course of schizophrenia who show low adherence to oral medication. The studies performed especially with LAI risperidone in patients with first episode or early stage of schizophrenia clearly indicated that this form of therapy could be effective and well tolerated also in this subgroup of patients with schizophrenia. Thanks to the familiar mother molecule (paliperidone is 9-OH risperidone, which is the active metabolite of risperidone) data concerning efficacy and tolerability of LAI risperidone in first-episode schizophrenia, patients could be applicable for paliperidone palmitate as well. Combined benefits of AP2G characteristics with an assured route of administration raise a question whether LAI AP2G should be recommended also in first-episode patients [46, 47]. Instead of foreknowing/guessing whether patients will accept LAI therapy or not, psychiatrists should offer this form of treatment as a routine choice to all appropriate patients with schizophrenia, including first-episode subjects. Selection between long-term injectable therapy and oral medication should be based on educational and therapeutic dialogue of the psychiatrist and the patient who can then in turn discuss the potential benefits and disadvantages of the proposed therapeutic strategy [48, 49].

Approximately 80% of patients with a first episode of schizophrenia reach symptomatic remission after antipsychotic therapy. However, within two years most of them relapse mainly due to low insight into the illness and non-adherence to oral medication. Therefore, although the formal data available are limited many experts recommend prescribing long-acting second-generation antipsychotics (especially LAI risperidone or alternatively paliperidone palmitate) in the early stages of schizophrenia, particularly in patients who benefited from the original oral molecule in the past and agree to receive long-term injection treatment. Early application of long-acting injectable second-generation antipsychotics can significantly reduce the risk of relapses in future and thus improve not only social and working potential of patients with schizophrenia but also their quality of life.

## Figures and Tables

**Table 1:** Survey of studies on LAI risperidone in first-episode schizophrenia patients.

Author, year	Study design	Duration of schizophrenia	*N*	Comparator	Result
Parellada et al., 2005 [39]	Six-month open study	Median one year, no more than 3 years	382	0	73% patients completed the study, statistically significant decrease of PANSS; 40% patients achieved at least 20% reduction of total PANSS score.
Malla et al., 2006 [44]	Two-year open study	Up to 3 years	15	Oral AP2G	More significant reduction of total PANSS score versus oral risperidone.
Emsley et al. 2009 [30]	Two-year open study	First episode	50	0	72% patients completed the study; 78% of them reached at least 50% reduction of symptoms; remission persisted for two years in 62% patients [42].
